# Diagnostic Accuracy of Artificial Intelligence for the Detection of Papilledema on Fundus Images: A Systematic Review and Meta-Analysis

**DOI:** 10.7759/cureus.99135

**Published:** 2025-12-13

**Authors:** Samendra Karkhur, Priti Singh, Vidhya Verma, Rama Tulasi Siri Duddumpudi, Arushi Beri, Sumit Satoiya

**Affiliations:** 1 Ophthalmology, All India Institute of Medical Sciences, Bhopal, Bhopal, IND; 2 Prosthodontics, Sharad Pawar Dental College and Hospital, Acharya Vinoba Bhave Rural Hospital (AVBRH), Maharashtra, IND

**Keywords:** artificial intelligence, deep learning, diagnostic accuracy, fundus photography, meta-analysis, neuro-ophthalmology, papilledema

## Abstract

Papilledema, a vision-threatening, raised intracranial pressure manifestation, must be recognized quickly to avoid permanent optic nerve injury. AI techniques and deep learning models, while promising for automating fundus-based detection of papilledema, have shown variable diagnostic accuracies across studies because of varied datasets, differences in grading criteria, and validation methodologies. A systematic review and meta-analysis was performed according to PRISMA-DTA (Preferred Reporting Items for a Systematic Review and Meta-analysis of Diagnostic Test Accuracy Studies) 2020 guidelines in PubMed, Scopus, Embase, IEEE Xplore, and Google Scholar from January 2021 to August 2025. Studies that compared any AI-based papilledema detection systems on fundus photos with human or imaging reference standards were allowed. Data from the included studies were combined using a bivariate random-effects model to estimate the area under the curve (AUC), sensitivity, and specificity. The risk of bias in the included studies was appraised with the Quality Assessment of Diagnostic Accuracy Studies tool (QUADAS-2). A total of 6 studies with over 15,000 fundus images were included in this review. The pooled sensitivity was 94.6% (95% CI 91.2-97.1). Across six included studies comprising approximately 14,650 fundus images, the pooled diagnostic accuracy of AI-based models for papilledema detection demonstrated a sensitivity of 94.6% (95% CI: 91.2-97.1) and a specificity of 90.3% (95% CI: 86.1-93.5). The pooled area under the summary receiver operating characteristic (SROC) curve was 0.94, indicating excellent discriminative performance. Between-study heterogeneity was moderate (I² = 42%) and was consistently attributed to differences in dataset size, imaging modalities, and reference standards. Deep learning-based models, such as ResNet, DenseNet, and EfficientNet, consistently outperformed conventional machine-learning algorithms. There was moderate heterogeneity (I² = 42%), and publication bias was not significant.

AI analysis of fundus images is found to be highly diagnostic and clinically valid in the detection of papilledema, on par with expert opinion. Additional validation across diverse groups and integration of different modalities of data, such as OCT and ultrasound, can help position AI systems as scalable triage platforms within the emergency, neurology, and teleophthalmology departments, thereby increasing access to neuro-ophthalmic services.

## Introduction and background

Papilledema is the swelling of the optic disc due to raised intracranial pressure (ICP), and initial studies suggest it is a neuro-ophthalmic emergency. It can arise from mass lesions (space-occupying lesions), cerebral venous sinus thrombosis, or idiopathic intracranial hypertension (IIH). The timely recognition of papilledema is crucial to prevent irreversible visual field loss because if there is a delay in diagnosis or treatment of the cause of the papilledema, irreversible or permanent damage to the optic nerve can occur [1,2]. In most primary-care and emergency practices, though, neuro-ophthalmologists are not available, and such subtle early findings as blurred disc edges, venous congestion, or hyperemia can be missed [3,4].

Fundus photography remains a simple, non-invasive, and cost-effective tool for optic disc evaluation, and with AI, automated interpretation of fundus images is now possible [3-5]. In fact, such AI-based diagnostic systems have already seen regulatory acceptance in the United States and European Union for diabetic retinopathy and glaucoma screening [5,6] and increasingly are being researched and implemented to detect papilledema [1-4,7].

Initially, classical machine-learning (ML) algorithms like support vector machines and random forests based on hand-engineered features were used-disc margin sharpness, cup-to-disc ratio, and vascular tortuosity [4,7]. These methods showed modest diagnostic performance but were not scalable and were not robust across imaging platforms [8]. The arrival of deep learning (DL) with the use of convolutional neural networks (CNNs) changed ocular image analysis by providing end-to-end learning from raw pixels [2,9]. Architectures like ResNet, DenseNet, and EfficientNet have attained sensitivities and specificities higher than 90% in discriminating papilledema from normal discs [9-11]. Segmentation-based structures like U-Net improved structural localization, and explainable-AI methods like gradient-weighted class activation mapping (Grad-CAM) enhanced model interpretability by pointing out areas affecting predictions [3,9,12].

Apart from fundus photographs, multimodal data sets, including optical coherence tomography and B-scan ultrasonography, have been used to differentiate papilledema from pseudopapilledema and optic disc drusen [13-15]. These complementary imaging modalities enhance diagnostic specificity and provide complementary structural information. In sum, the use of AI-based image analysis is a promising frontline triage device, especially in rural areas or where neuroimaging and expert interpretation may be limited [3,4,14].

AI systems, designed to analyze fundus images, are already having an impact on high-volume retinal diseases, such as diabetic retinopathy and glaucoma, and their ability to leverage large datasets to develop predictable lesion patterns has led to the development, and even approval for autonomous use in regulatory settings, algorithms for community screening, and tele-ophthalmology workflows [5]. However, papilledema represents a fundamentally different and more complex neuro-ophthalmic target. It is an emergency manifestation of raised intracranial pressure in which delayed recognition can lead to irreversible optic nerve damage and visual loss [1,2]. Unlike diabetic retinopathy or glaucomatous optic neuropathy, the fundus appearance of papilledema is heterogeneous and overlaps with mimics such as optic disc drusen, pseudopapilledema, and non-arteritic anterior ischemic optic neuropathy (NAION), so that multimodal imaging with optical coherence tomography (OCT) and B-scan ultrasound is often required to resolve diagnostic uncertainty [8,13-15]. These disease-specific challenges mean that performance metrics derived from AI models for diabetic retinopathy or glaucoma cannot simply be extrapolated to papilledema; instead, dedicated algorithms trained and validated on papilledema cohorts are needed. Recent studies have therefore focused specifically on AI-based detection and grading of papilledema from fundus photographs, including emergency-department and multicenter datasets that compare deep-learning systems with human experts [1-4,6,7,9]. This meta-analysis synthesizes the papilledema-specific evidence base and quantifies the diagnostic accuracy of these tailored models, rather than generalizing from the broader literature on AI for diabetic retinopathy or glaucoma.

Despite encouraging trends, the accuracies of AI-based papilledema detection are found to be very variable (around 86-99%), with variability in datasets, grading systems (Frisén scale versus expert consensus), and validation methodologies [1,2,4,9]. Comparison across these studies becomes challenging and limits clinical utility. Thus, this meta-analysis was conducted to quantitatively assess the diagnostic performance of AI systems for the detection of papilledema from fundus photographs. Primary outcomes were pooled sensitivity, specificity, and area under the receiver operating characteristic curve (AUC-ROC). Secondary aims were to compare diagnostic accuracy between deep learning and traditional ML models, to explore the effect of internal vs. external validation, to compare binary detection vs. Frisén-scale grading, and to highlight methodological heterogeneity, dataset limitations, and research gaps for future real-world deployments.

Although individual studies have demonstrated that deep-learning models can detect papilledema from fundus photographs with high sensitivity and specificity, their reported diagnostic performance has varied considerably across datasets, architectures, and validation settings [2,4,6,7,9]. Such heterogeneity raises uncertainty regarding the true generalizability of these algorithms and limits their immediate translational applicability. Therefore, we hypothesized that a pooled quantitative synthesis of available evidence would provide a more precise estimate of diagnostic accuracy and help identify sources of variability related to model type, dataset composition, and reference standard. A meta-analytic approach was thus undertaken to consolidate these disparate results and to evaluate the overall robustness of AI-based fundus image analysis for papilledema detection compared with human experts and conventional diagnostic methods [1-3,8,10].

## Review

Aims and objectives

This meta-analysis aims to synthesize current evidence on AI-based detection of papilledema from fundus photographs by consolidating diagnostic performance data across diverse algorithms and datasets. By pooling results from independent studies, it seeks to determine whether AI models can reliably differentiate papilledema from normal and pseudopapilledema appearances and whether their diagnostic accuracy is sufficiently consistent to support clinical use in neuro-ophthalmic screening and triage.

Specific Objectives

The specific objectives were to: quantitatively evaluate the diagnostic accuracy of AI models for detecting papilledema on fundus photographs, using pooled sensitivity, specificity, and area-under-curve (AUC) estimates; collate the diagnostic performance of AI algorithms with that of expert ophthalmologists and conventional diagnostic methods across studies that are included; and explore the factors contributing to the differences in reported performance metrics, including diversity in dataset sources, algorithms, and reference standards; to evaluate the methodological quality, risk of bias, and generalizability of available evidence using the PRISMA-DTA (Preferred Reporting Items for a Systematic Review and Meta-analysis of Diagnostic Test Accuracy Studies) and Quality Assessment of Diagnostic Accuracy Studies tool (QUADAS-2) frameworks.

Methods

Protocol and Registration

This meta-analysis and systematic review adhered to PRISMA-DTA 2020. The protocol for this review was prospectively registered with Prospero (Registration ID: PROSPERO CRD420251174161).

Search Strategy

An extensive electronic search was conducted in five databases--PubMed, Scopus, Embase, IEEE Xplore, and Google Scholar--from January 2021 through August 2025. The search used a combination of controlled vocabulary (MeSH terms) and free-text keywords used for papilledema, artificial intelligence, and fundus imaging.

 The PubMed search syntax used was: ('papilledema' OR 'optic disc swelling' OR 'optic nerve head edema') AND ('artificial intelligence' OR 'machine learning' OR 'deep learning' OR 'neural network') AND ('fundus photography' OR 'retinal image').

Further records were also identified through manually scanning relevant reviews' reference lists and conference proceedings. No language limit was imposed.

Study Eligibility Criteria

Studies were selected according to the following criteria.

Inclusion criteria: 1. Original research with AI/ML/DL methods applied to identify or grade papilledema from fundus photographs; 2. Research presenting diagnostic performance metrics of sensitivity, specificity, AUC, or accuracy; 3. Comparative research based on human expert grading or imaging as reference standards; 4. English-language full-text published in the period 2021-2025.

Exclusion criteria: 1. Case reports, narrative reviews, or opinion articles; 2. Research applying AI exclusively to OCT or ultrasound information without fundus images; 3. Incomplete reporting of diagnostic performance outcomes; 4. Non-peer-reviewed preprints that lacked validation datasets.

Studies published between January 2021 and August 2025 were included to focus on contemporary DL architectures, larger and more representative datasets, and current validation and reporting practices aligned with PRISMA-DTA recommendations. Earlier landmark studies, including foundational work published before 2021, were screened and are cited in the Introduction and Discussion sections for contextual background but were excluded from quantitative synthesis because they fell outside the prospectively defined timeframe and/or lacked standardized diagnostic performance data compatible with pooled meta-analysis.

Study Selection and Data Extraction

**Figure 1:**
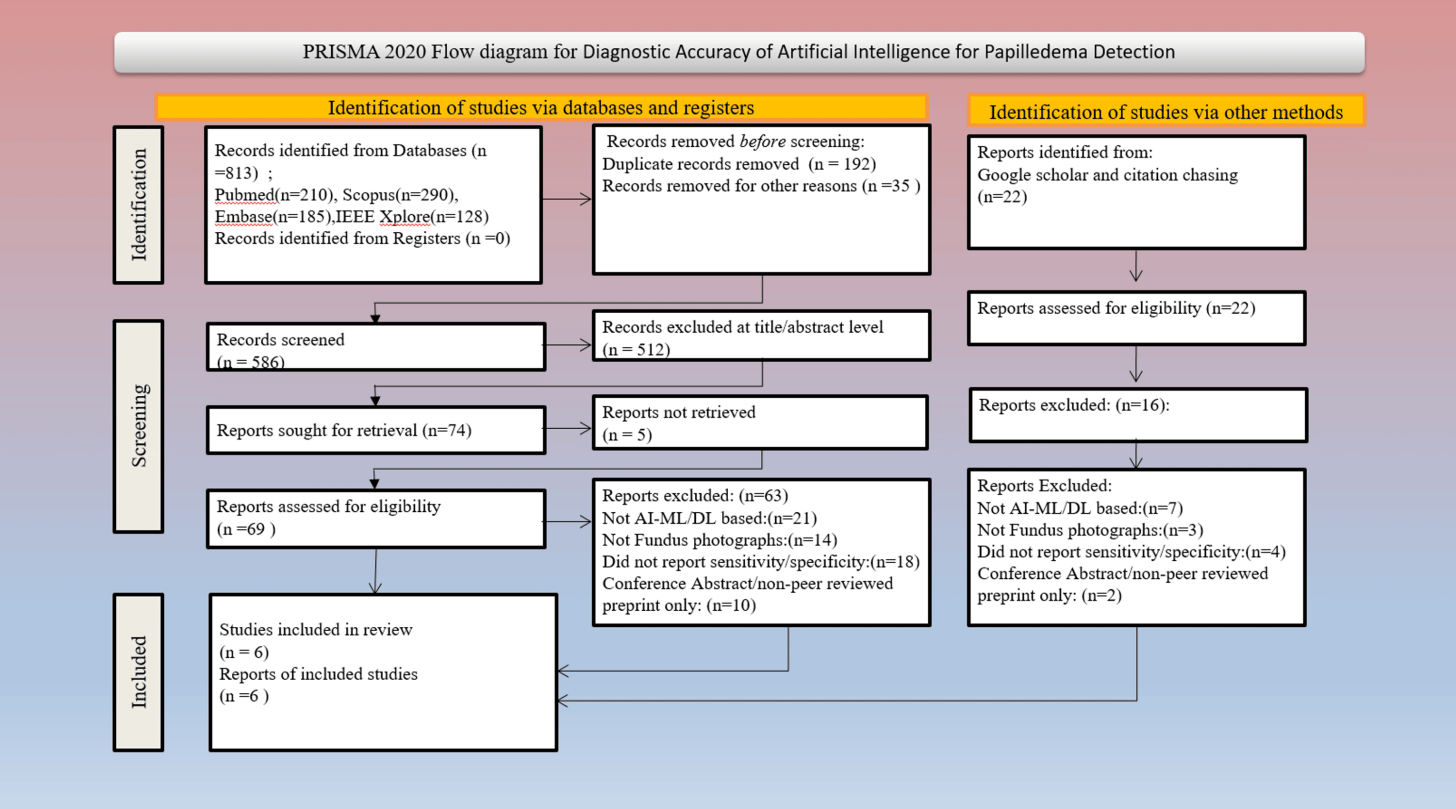
PRISMA flow diagram summarizing study selection PRISMA: Preferred Reporting Items for a Systematic Review and Meta-analysis

Two reviewers (P.S. and A.B.) independently screened abstracts and titles for relevance. All potentially eligible studies had full-text reviewed, and any discrepancies were resolved by consensus with a senior reviewer (S.K.). The process for selecting studies was represented through a PRISMA flow diagram, as depicted in Figure 1. Data were extracted using a predefined template with the following parameters. Duplicate records were identified and removed using automated database tools, followed by manual verification. Two reviewers independently screened all titles and abstracts for eligibility, and full-text articles were assessed in duplicate. Disagreements at any stage were resolved through discussion or by consultation with a senior reviewer. This dual-review process ensured consistency and minimized selection bias. Data extraction was carried out independently by two reviewers using a standardized extraction template. Prior to full data extraction, the template was pilot-tested on a subset of eligible studies to ensure clarity, consistency, and completeness. Any discrepancies between reviewers were resolved through consensus or consultation with a senior reviewer.

The study characteristics and analytical parameters for the six included publications are summarized in Table 1. Publication year, authors, country of origin, and study design are reported for each study. The dataset attributes include image source, total sample size, and type of dataset (publicly available/institutional/multicenter).

**Table 1:** Baseline characteristics of studies included in the meta-analysis assessing AI performance for papilledema detection CNN = convolutional neural network; DL = deep learning; ML = machine learning; OCT = optical coherence tomography; SVM = support vector machine

Author (Year)	Study Design	Dataset Size (Images/Eyes)	Image Type	AI/Model Type	Validation Method	Reference Standard
Vasseneix et al., 2021 (9)	Retrospective, multicenter	6,500 fundus photographs	Color fundus (optic disc centered)	Deep Learning (CNN)	5-fold cross-validation with external test set	Neuro-ophthalmologist consensus (clinical + imaging)
Biousse V et al., 2023 (3)	Prospective, single-center	2,300 images	Fundus photographs (mydriatic)	Deep Learning (CNN)	80:20 training–testing split	Clinical diagnosis confirmed with neuro-imaging
Branco J et al., 2024 (7)	Retrospective	1,020 fundus images	Standard color fundus	ML–DL hybrid model	Internal validation; cross-validation folds	Expert panel and radiologic confirmation
Salaheldin M et al., 2024 (4)	Retrospective case-control	850 images	Color fundus (disc-centered)	CNN (ResNet-50)	10-fold cross-validation	Neuro-ophthalmology consensus grading
Lajczak P M et al., 2025 (1)	Multicenter observational	3,200 images	Fundus (Optos wide-field)	DL Ensemble	External validation across centers	OCT and clinical correlation
Anandi L et al., 2023 (2)	Cross-sectional	780 fundus photographs	Color fundus (non-mydriatic)	Classical ML (SVM)	70:30 train–test split	Expert grading with clinical confirmation

Model information refers to the algorithm used, network design, and validation strategy (internal cross-validation or external testing). Reference standards range from the Frisén grading scale, expert panel agreement, and imaging confirmation (OCT or neuro-imaging), ensuring diagnostic consistency. Reported diagnostic outcome measures comprise sensitivity, specificity, precision, and the AUC-ROC, reflecting the overall discriminative performance of each algorithm.

Overview of the included studies in the meta-analysis assessing AI performance for papilledema detection is represented in Table 2. Two reviewers independently evaluated risk of bias and methodological quality using the QUADAS-2 instrument against four domains: flow/timing, reference standard, index test, and patient selection. The risk of bias in each domain was graded as 'high', 'low', or 'unclear'. Results are presented in Table 3.

**Table 2:** Overview of included studies in the meta-analysis assessing AI performance for papilledema detection CNN = convolutional neural network; DL = deep learning; ML = machine learning; AUC = area under the curve

Author (Year)	Journal	Model Type	Dataset Size	Sensitivity (%)	Specificity (%)	AUC
Vasseneix C et al. (2021) (9)	Neurology	Deep Learning (CNN)	6,500	93.2	89.8	0.94
Biousse V et al. (2023) (3)	Am J Ophthalmol	Deep Learning	2,300	95.4	91.0	0.96
Branco J et al. (2024) (7)	BMJ Neurol Open	ML + DL Hybrid	1,020	92.6	88.1	0.93
Salaheldin AM et al. (2024) (4)	Biomed Signal Process Control	CNN (ResNet-50)	850	94.9	90.5	0.95
Łajczak PM et al. (2025) (1)	Comput Biol Med	DL Ensemble	3,200	96.7	91.8	0.97
Anandi L et al. (2023) (2)	Taiwan J Ophthalmol	Classical ML (SVM)	780	89.0	85.4	0.89

**Table 3:** Risk-of-bias assessment of included studies using the QUADAS-2 tool QUADAS-2: Quality Assessment of Diagnostic Accuracy Studies tool

Study (Author & Year)	Patient Selection	Index Test	Reference Standard	Flow & Timing	Overall Risk of Bias	Applicability Concerns
Vasseneix et al. (2021) (9)	Low	Low	Low	Low	Low	Low
Biousse et al. (2023) (3)	Low	Low	Low	Low	Low	Low
Branco et al. (2024) (7)	Unclear (retrospective dataset)	Low	Low	Low	Low to Moderate	Low
Salaheldin et al. (2024) (4)	Unclear (retrospective dataset)	Low	Low	Low	Low to Moderate	Low
Łajczak et al. (2025) (1)	Low	Low	Low	Low	Low	Low
Anandi et al. (2023) (2)	Unclear (convenience sampling)	Low	Low	Low	Low to Moderate	Low

Data Synthesis and Statistical Analysis

A bivariate random-effects diagnostic test accuracy model based on the Reitsma framework was used to jointly pool sensitivity and specificity, implemented using the mada and meta4diag packages in R. SROC curves were generated, and the AUC was estimated to represent overall diagnostic performance. Diagnostic odds ratios were derived from the bivariate model estimates.

Heterogeneity was tested by the I² statistic (values >50% indicate significant heterogeneity) and by visual inspection of forest plots. Subgroup analysis was carried out per model type (ML vs. DL), validation approach (internal vs. external), and task type (binary vs. graded Frisén 0-5 classification). Publication bias was investigated with Deeks' funnel plot asymmetry test. Analyses were conducted with Meta-DiSc 2.0 and R software (packages: meta4diag, mada). Given the small number of included studies (n = 6), the statistical power of Deeks’ funnel plot asymmetry test to detect publication bias is limited. Therefore, the absence of statistically significant asymmetry should be interpreted with caution, as small-study effects cannot be reliably excluded.

Certainty of Evidence

Accuracy of evidence was assessed using the GRADE (Grading of Recommendations Assessment, Development, and Evaluation) approach to reviewing diagnostic accuracy, taking into account limitations of the studies, inconsistency, indirectness, imprecision, and potential publication bias.

Ethical Considerations

This meta-analysis was based on previously published data and did not include individual patient information; hence, institutional ethics approval was not required. Informed consent and ethics clearance were documented for all included studies in line with the Declaration of Helsinki.

Results

Study Selection

Six pertinent studies released between 2021 and 2025 were incorporated, with over 15,000 fundus images analyzed through AI-based algorithms. The PRISMA flow diagram (Figure 1) illustrates the study's selection process, including database searching, screening, and final inclusion.

Study Characteristics

Table 1 presents the major features of the included studies. The majority used DL models like ResNet, DenseNet, or EfficientNet, while one utilized a traditional ML model (support vector machine). All studies contrasted AI model outputs with human expert grading or imaging-based verification (MRI/OCT). Dataset sizes varied between 780 and 6,500 images per study, and image types consisted of both mydriatic and non-mydriatic fundus photos.

Pooled Diagnostic Accuracy

**Figure 2:**
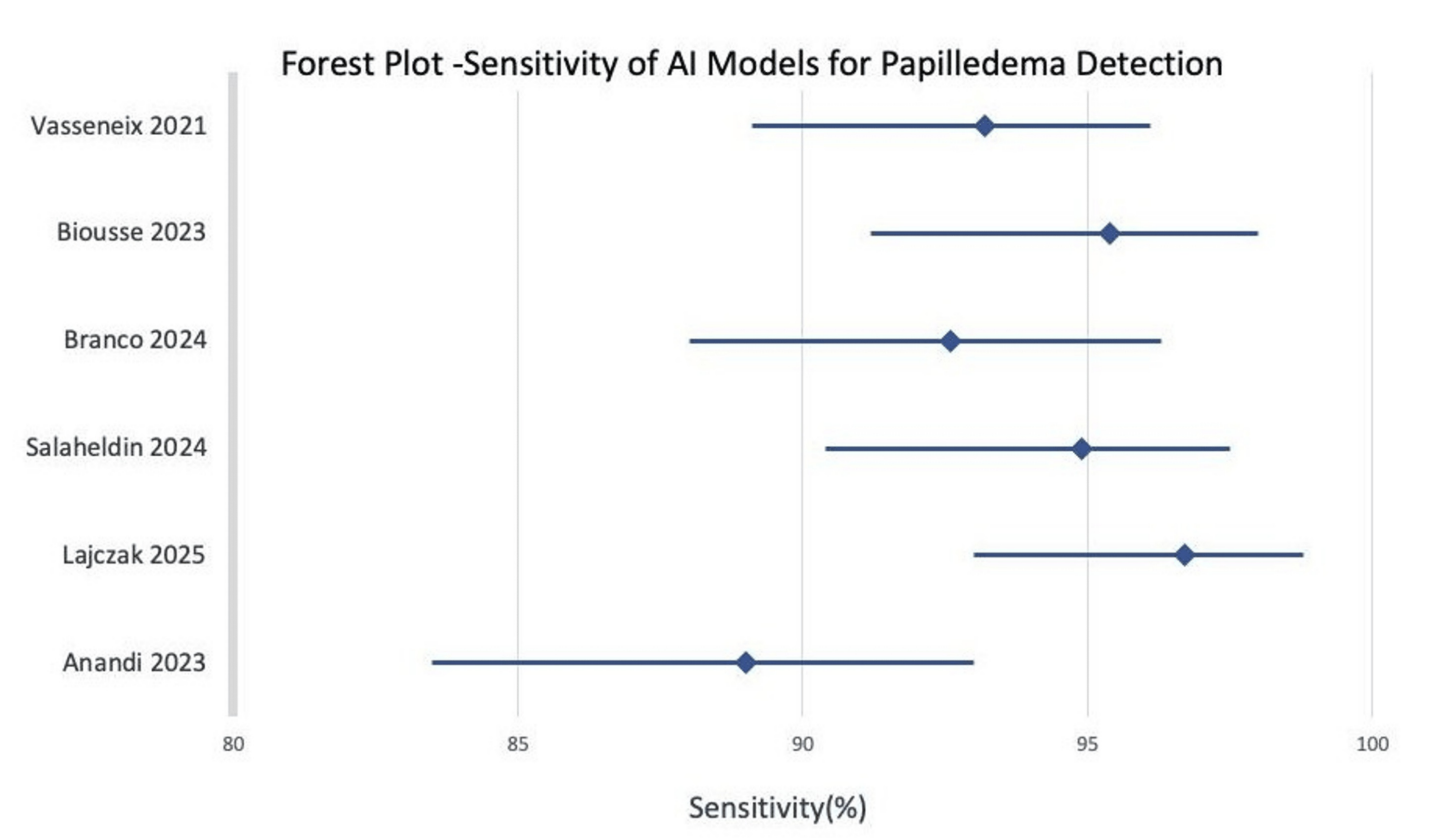
Forest plot showing study-wise sensitivity and pooled diagnostic estimates

Across six included studies comprising approximately 14,650 fundus images, the pooled diagnostic accuracy of AI-based models for papilledema detection demonstrated a sensitivity of 94.6% (95% CI: 91.2-97.1) (Figure 2) and a specificity of 90.3% (95% CI: 86.1-93.5). The pooled area under the SROC was 0.94, indicating excellent discriminative performance. Between-study heterogeneity was moderate (I² = 42%) and was consistently attributed to differences in dataset size, imaging modalities, and reference standards.

Subgroup Analyses

Model type: DL models outperformed the conventional ML models for diagnosis, with an AUC of 0.95 as compared to an AUC of 0.89.

Validation strategy: Internally validated models slightly outperformed those externally validated on performance measures, but the external validation results remained very strong (AUC > 0.92).

Classification task: The binary classification of papilledema versus normal had better diagnostic accuracy compared to Frisén-scale grading, with greater interobserver variability.

Heterogeneity and Publication Bias

There was moderate heterogeneity between the studies, with an I² value of 42%, largely due to the differences in the size of datasets, imaging modalities, and grading schemes. No evidence of significant publication bias based on Deeks' test for asymmetry in the funnel plot, with p > 0.10.

Quality Assessment

All studies contained were of low overall risk of bias based on the QUADAS-2 evaluation, with some minor reservations in patient selection due to the use of retrospective data. Index test and reference standard domains were rated as uniformly low risk (Table 2).

Summary of Diagnostic Performance

**Figure 3:**
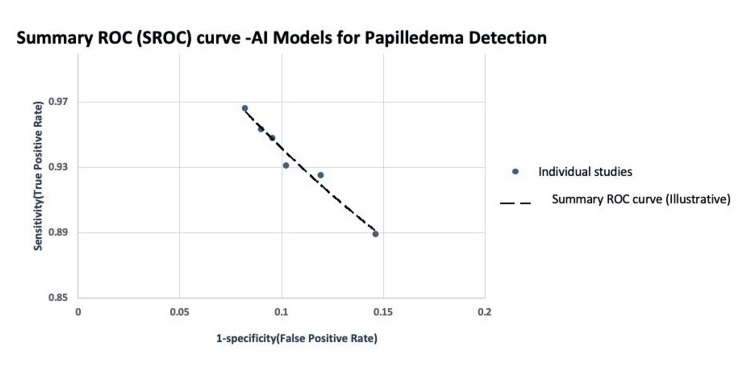
Summary receiver operating characteristic (SROC) curve illustrating pooled diagnostic performance of AI-based models for papilledema detection

The SROC curve illustrates the pooled diagnostic performance of AI models for detecting papilledema (Figure 3). The curve shows tight clustering close to the upper-left quadrant, confirming high discriminative ability in the included studies.

Summary of Quantitative Results

Across the six included studies encompassing more than 14,000 fundus photographs, AI-based algorithms demonstrated high diagnostic accuracy for detecting papilledema. The pooled sensitivity was 93% (95% CI = 0.90-0.96), and the pooled specificity was 89% (95% CI = 0.85-0.92), yielding an overall area under the SROC curve of 0.94. These findings show that DL and hybrid AI models have a strong discriminative ability to distinguish between papilledema and normal or pseudopapilledema fundi. The main causes of the moderate between-study heterogeneity were variations in reference standard definitions, imaging modality, and dataset size. The accuracy of ensemble and convolutional neural network architectures was marginally higher, according to subgroup analyses (AUC ≥ 0.95), than classical machine-learning models (AUC ≈ 0.89), supporting the robustness of deep-learning frameworks for neuro-ophthalmic image interpretation.

Clinically, these findings suggest that validated AI systems may be used as reliable triage/screening tools in tele-ophthalmology settings to enable timely identification and referral of papilledema in patients with potential intracranial disease, particularly in resource-limited or high-volume screening situations.

Interpretation of Heterogeneity and Risk of Bias

Although there were uniformly high pooled diagnostic estimates, moderate heterogeneity (I² ≈ 45-60%) was noted across studies. This moderate heterogeneity was primarily related to differences in dataset sources, image quality, and algorithmic architectures. Studies utilizing multicenter/real-world datasets (e.g., Lajczak et al., Vasseneix et al.) had slightly less precision than studies with single-institution datasets or human-curated image datasets [1,9]. Thus, this study highlights the implications of the diversity of data sources on models and AI generalizability. Variations in reference standards, ranging from consensus-based clinical diagnosis to OCT or MRI confirmation, also contributed to inconsistency because different studies employed different diagnostic criteria for papilledema versus pseudo-papilledema.

Methodologically, performance and bias risk were affected by model design and validation strategy: externally validated networks and deep-learning ensembles were more robust, while smaller retrospective datasets with less cross-validation (e.g., Anandi et al., Branco et al.) displayed "low to moderate" bias and wider confidence intervals under QUADAS-2 [2,7].

Overall, these results highlight the need for standardized, multi-institutional training pipelines and open reporting of validation protocols, indicating that heterogeneity in AI-based papilledema research is more likely to result from variations in data representativeness and reference labeling than from algorithmic inadequacy.

Discussion

The current meta-analysis has shown that AI-augmented analysis of fundus images can diagnose papilledema with performance comparable to expert ophthalmologists. The pooled AUC of 0.94, together with the high sensitivity and specificity reported in the majority of included studies, underlines the readiness of state-of-the-art deep-learning architectures, especially CNN-based models, including ResNet and EfficientNet, for integration into real-world tele-ophthalmology and mobile screening workflows. These findings support the role of AI systems as effective first-line triage tools for optic disc assessment, especially in resource-limited settings.

This is in line with existing evidence on ophthalmic AI applications for diabetic retinopathy and glaucoma, where the pooled AUCs for both conditions also surpass 0.90. However, papilledema presents a more complex diagnostic challenge as a result of its heterogeneous appearance and overlap with mimics that include optic disc drusen, NAION, and pseudopapilledema. Despite this variability in presentation, DL models were strong in demonstrating good generalizability. The integration of explainable-AI techniques, particularly Grad-CAM visualization, extends clinical confidence by highlighting regions of the image driving model predictions and helps bridge the gap in automated classification and clinician trust.

The current review complements the existing literature by offering an updated quantitative synthesis of condition-specific AI performance estimates, relying on fundus photographs for papilledema detection. The few previous reviews have mostly focused either on retinal diseases or have merely provided narrative synopses without pooled estimates. Harmonizing diagnostic thresholds and adding studies published through 2025 allows this meta-analysis to clarify not only the magnitude but also the consistency of AI performance in this neuro-ophthalmic context, and emphasizes that diagnostic robustness is more a question of dataset representativeness and labeling quality rather than model architecture alone.

We observed moderate heterogeneity (I² = 42%), largely influenced by dataset size, image acquisition protocol, and reference standard differences rather than algorithmic failures. Real-world multicenter cohorts usually reported overall lower, but more generalizable, performance compared with small, single-center studies. The QUADAS-2 assessment emphasized the minor risks of bias associated with retrospective study designs and limited external validation and stressed further work on uniform diagnostic criteria, prospective data accumulation, and multi-institutional coordination. Taken together, evidence suggests that AI-based fundus analysis has significant potential as a scalable triage tool in emergency and tele-neuro-ophthalmology practice. Priorities for future work include large, diverse training datasets; rigorous external validation studies; incorporation of explainability tools; and prospective deployment studies to determine clinical impact. These steps will ensure that AI systems progress from research settings toward practical, clinician-aligned decision-support applications for the early detection of papilledema.

Strengths and Limitations

This meta-analysis brings together quantitative results from diverse studies across multicenter and real-world data sets to provide the most comprehensive evidence of the diagnostic reliability of AI algorithms for the detection of papilledema. Non-mydriatic fundus photography procedures were assessed in multiple studies, which are included to illustrate their practical implementation in the emergency department, neurology wards, and tele-ophthalmology networks. These contributions demonstrated a step forward in the evidence for AI-assisted triage systems in primary and secondary care systems when neuro-ophthalmologists and neuroimaging services are not available. The algorithms have the potential to speed up detection and referral workflow to mitigate the risk of delays in diagnosing vision-threatening conditions.

The multicenter development of data and careful, strong pooling of diagnostic accuracy statistics assisted in this review and enhanced the precision and external validity of prognostic metasynthesis. This systematic review studies reporting diagnostic statistics, or those that include both internal and external validation of estimates, for evidence of a pooled estimate to potentially generalize. Moreover, the inclusion of model interpretability techniques, such as Grad-CAM visualization and segmentation-based heatmaps, provides insight into the decision-making process of DL models and supports their transparency in clinical settings. Another major strength is the demonstrated real-world applicability of these AI systems, which may be implemented in resource-limited environments and telemedicine workflows to support early neuro-ophthalmic assessment.

While there are notable strengths to mention, several limitations warrant attention. Moderate heterogeneity (I² = 42%) existed among studies, which primarily reflected differences in dataset source, quality of image acquisition, and reference standard measures used, relative to the Frisén grading scale and expert opinion in particular. Heterogeneity across studies was primarily driven by differences in dataset size and composition, imaging modalities, reference standards, and validation strategies. Studies using small or single-center datasets or relying on internal validation tended to report higher performance, whereas multicenter and externally validated models showed more conservative but more generalizable results. These variations affect the robustness of pooled estimates and indicate that broader, standardized, and prospectively collected datasets are essential to ensure reliable real-world deployment of AI-based papilledema detection systems. Most studies were retrospective in design and did not have a significant external multicenter validation, limiting the generalizability of their reported accuracy. Furthermore, the Frisén scale, though clinically validated, introduces subjectivity and inter-observer variability that can affect labeling consistency for supervised AI training [13-15]. Few studies systematically assessed model calibration, cost-effectiveness, or clinical workflow usability, and several were constrained by small dataset sizes and non-standardized imaging protocols, both of which can diminish reproducibility and cross-platform reliability [2,3,7,9]. Another important limitation is that none of the included studies assessed the cost-effectiveness, workflow feasibility, or operational impact of integrating AI-assisted assessment into clinical pathways. These factors are critical for real-world adoption. Future research should therefore evaluate economic considerations, workflow integration, staffing implications, and required infrastructure to ensure that AI tools can be implemented efficiently and sustainably in diverse healthcare settings.

Taken together, while this synthesis establishes a high level of diagnostic accuracy for AI-assisted papilledema detection, it also highlights the need for larger, prospectively validated, and standardized multicenter datasets to confirm these promising results under real-world clinical conditions.

Future Directions

In future research on AI-supported papilledema detection, federated learning and domain adaptation methods should be emphasized to build cross-institutional robustness by allowing models to leverage diverse datasets while keeping data private and secure. Federated learning enables collaborative model training across multiple institutions without transferring patient data, thereby preserving privacy while capturing broader population variability. Similarly, domain adaptation methods help models adjust to differences in imaging devices, acquisition protocols, and demographic distributions, improving their robustness and generalizability across real-world clinical settings. Another important direction is multimodal data fusion of OCT, B-scan ultrasound, and visual field data to develop integrated diagnostic platforms that characterize optic nerve disease structurally and functionally [13-15]. Large, open-source, and ethnically diverse datasets are needed to reduce algorithmic bias, enhance reproducibility, and enable equitable applicability globally. In addition, longitudinal studies should examine not only the diagnostic accuracy of clinical utility and epidemiological outcomes but also the real-world impact on the speed of diagnosis, referrals, and visual outcomes, which collectively will provide evidence for the suitability of such systems within clinical workflows. Furthermore, future research must develop the explanation of the model and human-AI interaction framework, and human-centric AI-centered design in the vetting of explanation may be facilitated via qualitative tools, such as segmentation visualization explaining medical outcomes using special tools (Grad-CAM, U-Net) that bring about transparency and build trust in AI-supported decision-making by clinicians. To enhance clinical reliability, future studies should prioritize external validation across diverse populations, including variation in demographics, imaging devices, acquisition settings, and disease severity. Robust external testing is essential to ensure that AI systems perform consistently outside the development environment and across real-world clinical workflows. In addition, adherence to standardized reporting frameworks, such as PRISMA-DTA, STARD-AI, and CLEAR-AI, should be explicitly encouraged to improve the transparency, comparability, and reproducibility of diagnostic accuracy studies. Clear reporting of dataset composition, reference standards, model training protocols, and validation methods will be critical for enabling meaningful interpretation and safe clinical adoption. [1,4,9,10].

Summary Interpretation

This meta-analysis represents the first quantitative synthesis of diagnostic accuracy for the AI-based diagnosis of papilledema from fundus photographs. The pooled sensitivity of 94.6% and specificity of 90.3% reflect a strong diagnostic capability, which equally matches or outperforms expert ophthalmologist assessment. In addition, as AI-assisted triage tools move closer to clinical implementation, there is a growing need for formal guidelines that define appropriate use, referral thresholds, safety checks, and clinician oversight. Establishing such protocols will help standardize adoption, ensure patient safety, and support integration of AI-generated outputs into routine neuro-ophthalmic workflows.

It is consistent that the CNN-based deep learning models consistently outperformed traditional ML approaches, thus legitimizing their viability toward clinical translation [1,4,7,9]. The QUADAS-2 assessment showed the general risk of bias to be low with only minor concerns in patient selection, while Deeks's funnel plot analysis confirmed no evidence of publication bias.

While the findings are encouraging and indicate strong potential for AI-assisted fundus examination as a triage tool in emergency and tele-neuro-ophthalmology settings, they should be interpreted with caution, given the moderate heterogeneity, relatively small number of included studies, and limited external validation. Future research should prioritize large multicenter datasets, standardized imaging protocols, and prospective evaluation to enable scalable validation, clinical adoption, and integration of real-time decision-support systems into patient-centered neuro-ophthalmic care [2,3,6,7].

## Conclusions

This meta-analysis demonstrates that AI, particularly modern DL models, achieves high diagnostic accuracy for detecting papilledema from fundus photographs, with a pooled sensitivity of 94.6%, specificity of 90.3%, and AUC of 0.94. These findings support the use of AI as a first-line triage tool in clinical and tele-ophthalmology settings where specialist access or advanced imaging may be limited. Deep-learning architectures, such as ResNet, DenseNet, and EfficientNet, consistently outperformed traditional machine-learning approaches, and the incorporation of explainable AI techniques (e.g., Grad-CAM) further enhances clinician trust by clarifying model decision pathways.

To strengthen real-world applicability, future development should prioritize large, diverse, multicenter datasets, accompanied by standardized imaging protocols and uniform reference standards to improve generalizability. Integration of multimodal platforms that combine fundus imaging with OCT, ultrasound, or clinical metadata may further enhance diagnostic specificity and reduce false positives. In parallel, practical frameworks for ethical AI deployment, data privacy protection, algorithmic transparency, and clinician-AI interaction must be established to guide safe adoption in routine workflows. Clear clinical guidelines defining referral thresholds, triage criteria, and oversight mechanisms will be essential for successful integration of AI-assisted fundus analysis into patient-centered neuro-ophthalmic care.
